# Diagnosis of penile cancer with *ex vivo* fluorescence confocal microscopy using the Histolog® Scanner

**DOI:** 10.1111/bju.70239

**Published:** 2026-03-23

**Authors:** Xiaohu Zhang, Ricardo Almeida‐Magana, Larissa Sena Teixeira Mendes, Daniela Fleck Lavergne, Hussain Alnajjar, Greg Shaw, Alex Freeman, Asif Muneer, Aiman Haider

**Affiliations:** ^1^ Department of Surgery and Interventional Science University College London London UK; ^2^ Department of Urology University College London Hospitals NHS Foundation Trust London UK; ^3^ NIHR Biomedical Research Centre University College London Hospital London UK; ^4^ Department of Cellular Pathology University College London Hospitals NHS Foundation Trust London UK

**Keywords:** penile cancer, fluorescence confocal microscopy, *ex vivo* imaging, intraoperative diagnosis, Histolog Scanner

## Abstract

**Objectives:**

To evaluate the feasibility and diagnostic performance of *ex vivo* fluorescence confocal microscopy (FCM) using the Histolog® Scanner (SamanTree Medical SA, Lausanne, Switzerland) for the assessment of penile cancer (PeCa) specimens, and to compare FCM‐based diagnoses with standard formalin‐fixed, paraffin‐embedded (FFPE) histopathology.

**Patients and Methods:**

We conducted a single‐centre study including 12 patients with clinical or radiological suspicion of PeCa who underwent diagnostic or excisional biopsy between June 2022 and November 2023. Fresh biopsy specimens were stained with a nuclear fluorescent dye and scanned *ex vivo* with the Histolog Scanner. Digital images were retrospectively reviewed by two uropathologists and compared with conventional haematoxylin and eosin‐stained FFPE sections. The primary endpoint was diagnostic concordance between FCM and histopathology in detecting invasive squamous cell carcinoma (SCC); secondary endpoints included sensitivity, specificity, and overall feasibility of image acquisition.

**Results:**

A total of 29 FCM scans were obtained. Diagnostic image quality was achieved in 28/29 scans (96.6%). FFPE analysis confirmed invasive SCC in eight patients, differentiated penile intraepithelial neoplasia in three, and an atypical squamous proliferative lesion (ASPL) in one. FCM correctly identified tumour category in 11/12 patients, yielding an overall accuracy of 91.7% (95% confidence interval [CI] 61.5–99.8%). Sensitivity for invasive SCC detection was 87.5% (7/8; 95% CI 47.3–99.7%), and specificity was 100% (4/4; 95% CI 39.8–100%). One SCC was misclassified as ASPL on FCM. Median workflow time was ~5 min/specimen.

**Conclusions:**

*Ex vivo* FCM is a feasible, rapid imaging technique that enables high concordance with histopathology for the diagnosis of PeCa. This pilot study represents the first assessment of *ex vivo* FCM in penile malignancies and provides preliminary evidence supporting its potential role in intraoperative margin assessment. Larger prospective studies are required to confirm its diagnostic accuracy and clinical utility.

**Abbreviations**
ASPL: atypical squamous proliferative lesion
FCM: fluorescence confocal microscopy
FFPE: formalin‐fixed, paraffin‐embedded
FS: frozen section
H&E: haematoxylin and eosin
HPV: human papilloma virus
p (T) (N): pathological (T stage) (N stage)
PeCa: penile cancer
(d)PeIN: (differentiated) penile intraepithelial neoplasia
SCC: squamous cell carcinoma

## Introduction

Penile cancer (PeCa) is a rare malignancy with the age‐adjusted incidence of 0.3–2.1 per 100 000 in Western Europe and North America [1]. Although patients with localised disease generally have a good prognosis, those presenting with advanced or metastatic disease present a therapeutic challenge. A histopathological diagnosis is required before undertaking glansectomy, partial penectomy or more extensive total penectomy procedures as the differential diagnosis can be challenging [2, 3]. Performing a formal biopsy with review of formalin‐fixed, paraffin‐embedded (FFPE) sections leads to a diagnostic delay in an already time dependent tumour. These delays can lead to disease progression or result in more extensive surgery being undertaken on the primary lesion. Incorporating intraoperative frozen section (FS) analysis has been used for diagnosis and checking the margin status during penile preserving surgery [4]. However, the cost, time delays, and the requirement of expert pathologists with availability at short notice limits its widespread adoption.

Fluorescence confocal microscopy (FCM) is a novel digital pathology‐based alternative to FS analysis. This technique allows fast, high‐resolution imaging of fresh, unfixed tissue, mimicking the appearance of haematoxylin and eosin (H&E) stained slides while preserving the integrity of the specimen for downstream molecular or histological analysis. *Ex vivo* FCM is currently being investigated to guide detection of positive margins in various surgical oncology fields, including breast, lung, prostate, bladder, and paediatric malignancies, demonstrating promising concordance with conventional histopathology [5, 6, 7, 8]. Its clinical utility is well established in dermatological oncology, where it is routinely employed during Mohs micrographic surgery to assess surgical margins in basal cell carcinoma and squamous cell carcinoma (SCC), offering diagnostic performance comparable to that of FS analysis [7, 9, 10].

Despite these advantages, the application of FCM in PeCa remains largely unexplored. FCM could enable rapid differentiation between benign and malignant lesions, which can expedite clinical decision‐making. Moreover, its potential to delineate tumour margins in real‐time could provide critical information for surgical planning [11]. As a first step to evaluate this technology in PeCa, we conducted an exploratory study utilising the Histolog® Scanner (SamanTree Medical SA, Lausanne, Switzerland) to assess the diagnostic performance of FCM on suspected PeCa specimens. This study aimed to compare FCM‐based diagnoses with standard FFPE histopathology for PeCa biopsy and to generate a reference image library to facilitate clinical adoption of this technology.

## Patients and Methods

### Study Design and Patient Selection

This was a single‐centre proof‐of‐concept study conducted between June 2022 and November 2023. A total of 12 patients with clinical or radiological suspicion of PeCa undergoing diagnostic biopsy or surgical excision were included when the FCM system and trained personnel were available during the study period. All procedures were performed in accordance with institutional standard guidelines for FS with no change in the surgical procedure. Standard consent and biobank consent was obtained for surgery and FS analysis. Some eligible patients did not undergo FCM due to logistical constraints, including scanner availability and workflow limitations. No cases were excluded after scanning.

### Tissue Acquisition and FCM

Following surgical biopsy of large exophytic lesions, fresh tissue specimens were bisected using a surgical scalpel. This achieved a flat surface amenable to analysis via FCM using the Histolog Scanner.

Tissues were submerged in a fluorescent nuclear dye (Histolog Dip) according to manufacturer guidelines. The stained tissue was positioned with the flat surface in contact with the objective and minimal compression using a light weight was applied to ensure full contact. Tissues were scanned producing a high‐resolution image within 50 s. Each scan took <1 min. No intraoperative clinical decisions were based on the FCM findings. Samples were transported to the histopathology department for standard FFPE analysis.

### Image Analysis

All FCM images were stored digitally and reviewed retrospectively by two experienced uropathologists who were unaware of the results of the corresponding H&E sections at the time of image interpretation. The images were further compared to the corresponding H&E slides. Images were evaluated for overall quality (surface completeness, staining contrast, nuclear clarity), presence of tumour, histological subtype, margin status (positive/negative), and other pathological features such as keratinisation, mitotic activity, perineural invasion, or presence of PeIN. These variables were documented using a standardised reporting sheet. The objective of the analysis was to understand the characteristics of PeCa tissue analysed by FCM. FCM images were analysed retrospectively by uropathologists who were not involved in the original FFPE H&E reporting, ensuring independent assessment. FCM findings were subsequently compared with the corresponding FFPE H&E sections, which served as the reference standard.

### Data Collection and Outcome Measures

Demographic and clinical data including age, surgery type, pathological (p)T/pN stage, H&E biopsy diagnosis, FCM biopsy diagnosis, SCC grade (H&E) and human papilloma virus (HPV) status were recorded for each patient.

The primary outcome was diagnostic concordance between FCM and H&E analysis in detecting invasive SCC. Secondary outcomes included sensitivity and specificity of assessment using FCM.

### Statistical Analysis

Diagnostic concordance between FCM and formal H&E assessment was evaluated by comparing the histological diagnosis assigned by each method on a case‐by‐case basis. Concordance was defined as an exact match between FCM and H&E in terms of diagnostic category, including invasive SCC, differentiated penile intraepithelial neoplasia (dPeIN), and atypical squamous proliferative lesion (ASPL).

To assess the diagnostic performance of FCM in identifying invasive SCC specifically, sensitivity and specificity were calculated using standard definitions. Sensitivity was defined as the proportion of cases with SCC confirmed on H&E that were also identified as SCC by FCM. Specificity was defined as the proportion of non‐SCC cases on H&E that were correctly identified as non‐SCC by FCM. H&E diagnosis was used as the reference standard. Accuracy estimates were calculated using R version 4.5.0 (R Foundation for Statistical Computing, Vienna, Austria) with package ‘epiR’.

## Results

### Patient Characteristics

A total of 12 patients with suspected or confirmed PeCa were included. The median (range) age was 65.5 (35–81) years. All patients underwent biopsy or surgical excision (glansectomy, partial penectomy, or glans resurfacing), with pathological staging ranging from pTis to pT3. Lymph node involvement was observed in five patients (pN1–pN3). HPV status was available for 10 patients, of whom seven (70%) tested positive by either PCR or immunohistochemistry.

### The FCM Image Acquisition and Technical Feasibility

A total of 29 *ex vivo* FCM scans were obtained from fresh tissue samples using the Histolog Scanner. The average acquisition time was ~5 min/specimen. Image quality was considered sufficient for diagnostic assessment in 28 of 29 scans (96.6%), based on pre‐defined criteria including surface completeness, contrast, and absence of major artefacts (e.g., air bubbles or staining irregularities).

### Histopathological Findings and Diagnostic Concordance

**Fig. 1:**
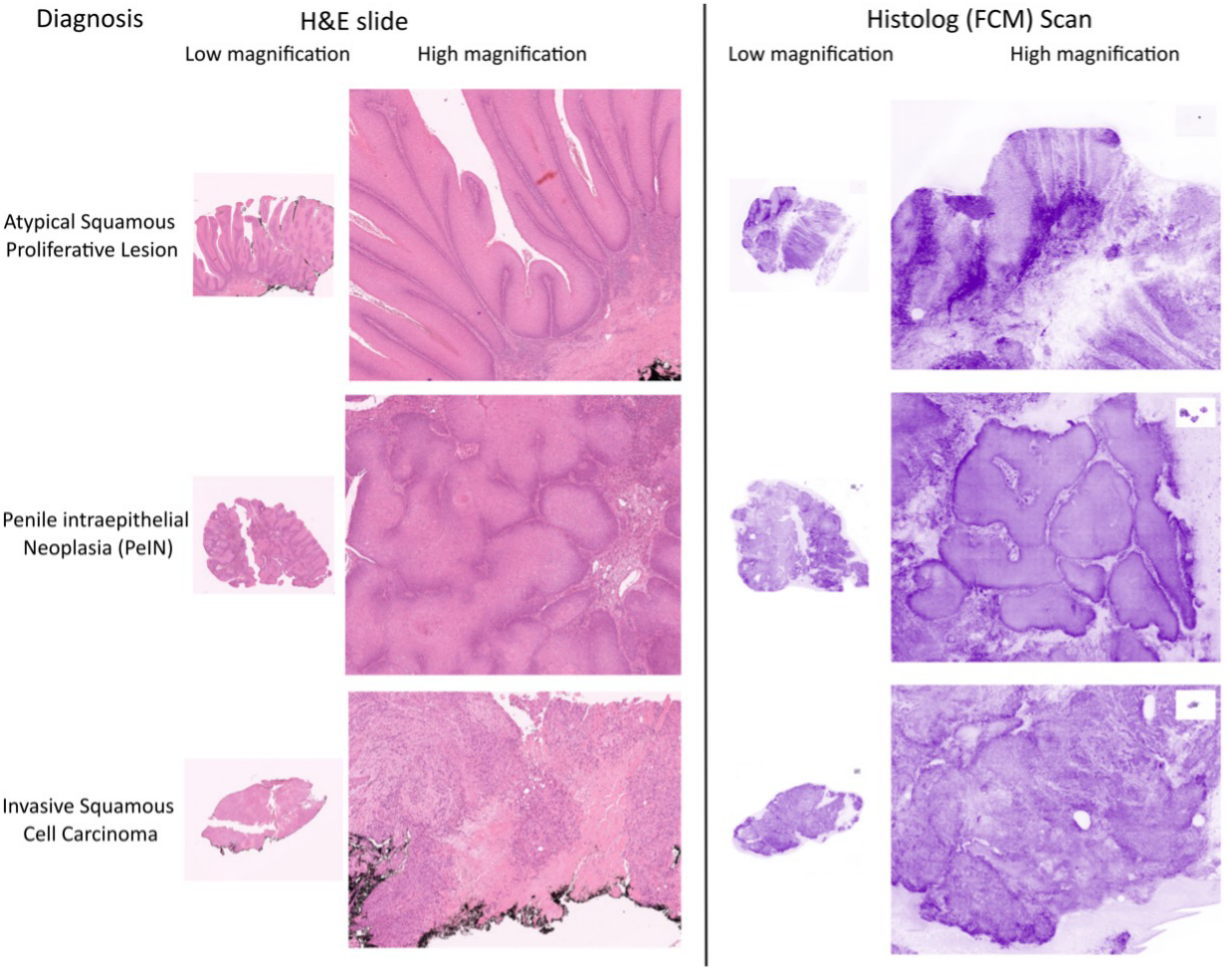
Comparative imaging of penile lesions on H&E histology and *ex vivo* FCM. Representative low‐ and high‐magnification images of three histological categories—atypical squamous proliferative lesion, PeIN, and invasive SCC—are shown side‐by‐side using conventional H&E staining (left) and corresponding Histolog FCM scans (right). The images illustrate the pathological characteristics with each modality and demonstrate the capacity of FCM to differentiate between various penile epithelial pathologies.

Histopathological evaluation of FFPE specimens confirmed invasive SCC in eight patients, dPeIN in three patients, and an ASPL in one patient. FCM correctly identified tumour presence in 11 of 12 patients, yielding a correctly classified proportion of 0.92 (95% CI 0.62–1.00). The single discordant case was diagnosed as SCC on H&E but diagnosed as ASPL on FCM. FCM demonstrated sensitivity of 0.88 (95% CI 0.47–1.00) and specificity of 1.00 (95% CI 0.40–1.00) for SCC detection (Tables 1 and 2; Fig. 1).

**Table 1:** Clinical characteristics of the study cohort.

Patient number	Age, years	Surgery type	H&E biopsy diagnosis	FCM biopsy diagnosis	SCC grade (H&E)	Final pT stage	Final pN stage	HPV status*
1	35	Glansectomy	SCC	SCC	3	pT3	pN3	Unknown
2	47	Biopsy	dPeIN	dPeIN	dPeIN	pTis	pNx	Positive
3	48	Partial penectomy	SCC	SCC	3	pT3	pN3	Positive
4	56	Glansectomy	SCC	SCC	3	pT2	pN1	Positive
5	56	Glansectomy	dPeIN	dPeIN	dPeIN	pT2	pN0	Unknown
6	64	Glansectomy	SCC	SCC	2	pT1a	pN0	Negative
7	67	Glans resurfacing	ASPL	ASPL	1	pT1a	pNx	Positive
8	70	Glansectomy	SCC	ASPL	4	pT1b	pN0	Positive
9	79	Partial penectomy	SCC	SCC	2	pT2	pN0	Negative
10	79	Partial penectomy	SCC	SCC	4	pT3	pNx	Positive
11	79	Partial penectomy	SCC	SCC	3	pT3	pN3	Positive
12	81	Partial penectomy	dPeIN	dPeIN	dPeIN	pT3	pN3	Negative

TNM staging based on the eighth edition of the Union Internationale Contre le Cancer/American Joint Committee on Cancer (UICC/AJCC). Final pathological stage was derived from subsequent definitive surgical specimens and may not correspond to the histology of the scanned biopsy specimen.

* HPV status tested with immunohistochemistry and/or PCR.

**Table 2:** A 2 × 2 diagnostic performance table comparing FCM and H&E for detection of invasive SCC.

	H&E = SCC	H&E ≠ SCC
FCM = SCC	7 (TP)	0 (FP)
FCM ≠ SCC	1 (FN)	4 (TN)
Sensitivity = TP/(TP + FN) = 7/8 = 87.5% Specificity = TN/(TN + FP) = 4/4 = 100%		

Sensitivity and specificity were calculated using FFPE H&E histopathology of the scanned specimen as the reference standard.

FN, false negative; FP, false positive; TN, true negative; TP, true positive.

## Discussion

This proof‐of‐concept study demonstrates the feasibility and diagnostic reliability of FCM in the evaluation of PeCa specimens. Compared to traditional FS analysis, FCM offers several technical advantages: image acquisition is fast, tissue preparation is minimal, and specimens remain intact for downstream histological or molecular studies [12]. In our cohort, FCM scans were completed within minutes, and the majority yielded diagnostically adequate images. Features relevant to malignant diagnosis, including epithelial stratification, keratinisation, and involvement of the epithelial stromal interface, were clearly visualised on FCM images.

One discordant case, in which invasive SCC was misclassified as ASPL on FCM, highlights current limitations in interpretive accuracy, particularly in borderline lesions. This underscores the need for standardised diagnostic criteria and training tailored to FCM interpretation in penile pathology. While FCM enables efficient tumour recognition, its capacity for reliable grading or detailed assessment of invasion remains limited due to inherent resolution constraints. FS in PeCa surgery to guide margin assessment has similar sensitivity [4, 13].

This is a novel study in its application of *ex vivo* FCM in PeCa cases, a rare malignancy with unique surgical and pathological considerations. To our knowledge, this is the first study to assess *ex vivo* FCM in this setting. The technique may be particularly valuable in scenarios where FS is unavailable or costly [14]. This is a common scenario in many centres where there is limited availability of expert pathologists or skilled technicians. Furthermore, the time delays between the sample acquisition and reporting (30–60 min) compounded by the requirement to transport the samples across facilities have made FS uptake limited [15]. Therefore, a faster method such as *ex vivo* FCM offers an attractive alternative.

Previous studies of FCM technology in PeCa have used *in vivo* imaging, with promising results [16]. However, this requires injection of intravenous fluorescein and offers a limited field of view with black and white images. This is in contrast to *ex vivo* FCM using the Histolog Scanner, where an image similar to H&E is produced, and the wide objective size allows several specimens to be analysed at the same time.

This technique opens the door to one stop clinics where patients with penile lesions requiring histopathological characterisation are booked in for diagnosis and treatment with minimal delay [17]. An additional advantage is tissue preservation for downstream evaluation with immunohistochemistry markers in case of uncertain histopathology diagnosis.

An important limitation of the present study is the lack of a direct comparison between *ex vivo* FCM and intraoperative FS analysis, which remains the reference standard for real‐time histological assessment in PeCa surgery. In this exploratory feasibility study, FS was not routinely performed for all cases, and FCM imaging was conducted in parallel without influencing intraoperative decision‐making. Therefore, FFPE histopathology was used as the reference standard. Future prospective studies directly comparing FCM with FS are warranted. Patients in this cohort underwent heterogeneous procedures, ranging from diagnostic biopsy to glans resurfacing and partial or total penectomy, resulting in variable tissue volume and sampling extent. This procedural heterogeneity may have influenced image acquisition and diagnostic performance and should be considered when interpreting the results. While FCM demonstrated high concordance in invasive SCC, its role in borderline lesions and PeIN remains to be fully defined. Subtle cytological atypia may be more challenging to interpret on confocal imaging, particularly as confocal morphology is less familiar to most pathologists compared with conventional H&E sections. Larger validation studies and training will be required to better define its diagnostic boundaries. Other limitations include the retrospective and exploratory nature of the image analysis and the small sample size. As this was the first exploration of this technology in this field our objective was to lay the groundwork for future studies. Nevertheless, the high level of diagnostic agreement and operational feasibility observed here provide a strong rationale for further prospective evaluation. Future studies should explore real‐time intraoperative implementation, interobserver reproducibility, and integration with digital and artificial intelligence‐assisted pathology platforms.

## Conclusion

Our findings indicate that *ex vivo* FCM is a feasible and potentially valuable tool for the evaluation of fresh PeCa specimens. Diagnostic concordance between FCM and conventional histopathology was high. This study represents the first assessment of FCM in the context of penile malignancies. While the results are promising, they should be interpreted with caution given the limited sample size and retrospective image review. Further validation in larger, prospective cohorts is necessary to establish the clinical utility of FCM in real‐time intraoperative diagnosis and surgical margin assessment in PeCa.

## Funding

No specific funding was received for this study.

## Disclosure of Interests

Subsequent to the acceptance of this article Prof Shaw has taken up a paid medical consultancy for SamanTree and is now a shareholder. All remaining authors declare that they have no conflicts of interest relevant to this work. [Correction added after first online publication on 21 May 2026: This section has been revised in this version.]
